# Functional properties and structural characterization of rice δ^1^-pyrroline-5-carboxylate reductase

**DOI:** 10.3389/fpls.2015.00565

**Published:** 2015-07-28

**Authors:** Giuseppe Forlani, Michele Bertazzini, Marco Zarattini, Dietmar Funck, Milosz Ruszkowski, Bogusław Nocek

**Affiliations:** ^1^Laboratory of Plant Physiology and Biochemistry, Department of Life Science and Biotechnology, University of FerraraFerrara, Italy; ^2^Plant Physiology and Biochemistry, Department of Biology, University of KonstanzKonstanz, Germany; ^3^Synchrotron Radiation Research Section, Macromolecular Crystallography Laboratory, National Cancer Institute, Argonne National Laboratory, ArgonneIL, USA; ^4^Biosciences Division, Argonne National Laboratory, ArgonneIL, USA

**Keywords:** proline synthesis, P5C reductase, enzyme properties, substrate ambiguity, cation and anion effects, product inhibition, oligomeric structure

## Abstract

The majority of plant species accumulate high intracellular levels of proline to cope with hyperosmotic stress conditions. Proline synthesis from glutamate is tightly regulated at both the transcriptional and the translational levels, yet little is known about the mechanisms for post-translational regulation of the enzymatic activities involved. The gene coding in rice (*Oryza sativa* L.) for δ^1^-pyrroline-5-carboxylate (P5C) reductase, the enzyme that catalyzes the second and final step in this pathway, was isolated and expressed in *Escherichia coli*. The structural and functional properties of the affinity-purified protein were characterized. As for most species, rice P5C reductase was able to use *in vitro* either NADH or NADPH as the electron donor. However, strikingly different effects of cations and anions were found depending on the pyridine nucleotide used, namely inhibition of NADH-dependent activity and stimulation of NADPH-dependent activity. Moreover, physiological concentrations of proline and NADP^+^ were strongly inhibitory for the NADH-dependent reaction, whereas the NADPH-dependent activity was mildly affected. Our results suggest that only NADPH may be used *in vivo* and that stress-dependent variations in ion homeostasis and NADPH/NADP^+^ ratio could modulate enzyme activity, being functional in promoting proline accumulation and potentially also adjusting NADPH consumption during the defense against hyperosmotic stress. The apparent molecular weight of the native protein observed in size exclusion chromatography indicated a high oligomerization state. We also report the first crystal structure of a plant P5C reductase at 3.40-Å resolution, showing a decameric quaternary assembly. Based on the structure, it was possible to identify dynamic structural differences among rice, human, and bacterial enzymes.

## Introduction

Among proteinogenic amino acids, proline plays an important role in protein structure, uniquely contributing to backbone folding and stability (Ge and Pan, 2009). Moreover, in most plants (Verbruggen and Hermans, 2008) and microorganisms (Empadinhas and da Costa, 2008; Takagi, 2008), a rapid and reversible increase of the intracellular concentration of free proline to high levels has been shown in response to either osmotic, oxidative, or temperature stress (Verslues and Sharma, 2010), implying a role in stress tolerance and osmoregulation (Szabados and Savouré, 2010; Hayat et al., 2012), redox balance (Liang et al., 2013), and apoptosis (Monteoliva et al., 2014). More recently, both the level of free proline and proline metabolism in plants were also hypothesized to influence the transition to flowering (Mattioli et al., 2009), as well as pollen and embryo development (Lehmann et al., 2010; Funck et al., 2012).

Although not conclusively analyzed experimentally (Funck et al., 2008), two metabolic routes leading to proline production have been proposed in higher plants. Under high nitrogen availability, the synthesis seems to proceed mainly through ornithine, an intermediate in the biosynthesis and degradation of arginine, which is converted to δ^1^-pyrroline-5-carboxylate (P5C) by a pyridoxal-dependent ornithine-δ-aminotransferase (da Rocha et al., 2012). Conversely, under osmotic stress conditions and/or nitrogen starvation P5C is synthesized from glutamate by P5C synthetase (Kavi Kishor et al., 1995; Turchetto-Zolet et al., 2009). The two pathways share the last reaction, in which P5C is reduced to proline by P5C reductase (EC 1.5.1.2). Although both P5C synthetase (Székely et al., 2008) and P5C reductase (Verbruggen et al., 1993) transcripts are induced under osmotic stress conditions, only the former is believed to represent a rate-limiting step (Kesari et al., 2012). In the absence of a functional P5C reductase both routes for proline biosynthesis are blocked, and no alternative pathway has been described. Consistently, null mutations of P5C reductase are embryo-lethal (Funck et al., 2012) and specific inhibitors of P5C reductase exert phytotoxic effects (Forlani et al., 2007), and may thus represent new active principles for weed control (Forlani et al., 2008).

As P5C reductase occurs at the converging point of these two anabolic pathways, it should be subjected to fine regulation, even though it might not represent a rate-limiting step under most conditions. Indeed, when P5C reductase protein levels and intracellular proline concentrations were measured in different tissues and in osmotically stressed seedlings, data were not in agreement with the corresponding mRNA levels (Hua et al., 1997). A complex pattern of regulation was postulated, in which differential mRNA stability, degree of polysome association and 5′UTR effects on translation efficiency seem to play a role (Hua et al., 2001). Yet, trans-acting factors that can bind to the *P5C reductase* promoter region or mRNA have not been identified. Moreover, a translation inhibition of *Arabidopsis thaliana* P5C reductase was found under stress conditions (Hua et al., 2001), a result that seems inconsistent with a role in stress-induced proline accumulation. The occurrence of post-translational regulative mechanisms was also proposed, but poorly investigated. In fact, plant P5C reductase has been purified only from a few plant species, such as barley (Krueger et al., 1986), soybean (Chilson et al., 1991), and spinach (Murahama et al., 2001). These enzymes showed substrate ambiguity, being able to use either NADH or NADPH as the electron donor, even if the NADH-dependent activity was inhibited by equimolar concentrations of NADP^+^ (Szoke et al., 1992). Moreover, a twofold stimulation of the NADH-dependent reaction by 100 mM KCl or 10 mM MgCl_2_ was reported for partially purified pea P5C reductase (Rayapati et al., 1989), whereas the two isozymes purified from spinach were on the contrary inhibited by NaCl (100–500 mM) and MgCl_2_ (10–100 mM) when assayed using NADPH as the co-factor (Murahama et al., 2001).

Recently, we isolated and characterized P5C reductase from suspension-cultured cells of *A. thaliana*, where a single gene is present (Verbruggen et al., 1993; Funck et al., 2012). The purified protein was able to use either NADPH or NADH as the electron donor, with contrasting affinities, and maximum reaction rates. The presence of equimolar levels of NADP^+^ completely suppressed the NADH-dependent activity, whereas the NADPH-dependent reaction was only mildly affected. Proline inhibited only the NADH-dependent reaction. At physiological levels, increasing concentrations of salt steadily inhibited the NADH-dependent activity, but were stimulatory of the NADPH-dependent reaction (Giberti et al., 2014). These properties suggest a complex regulation of enzyme activity by the redox status of the pyridine nucleotide pools, and the levels of proline and chloride in the cytosol. However, also due to the above inconsistencies in the literature, it was not possible to conclude whether these features are shared or not by all plant P5C reductases. Similarly, although a clear-cut preference for NADPH was evident, it was unclear whether NADH could sustain at least in part the rate of P5C reduction inside the plant cell under either physiological or stress conditions.

Besides the lack of a detailed biochemical characterization of the enzyme in a diverse array of plants, our knowledge of post-translational mechanisms regulating the activity of plant P5C reductase is hampered also by the unavailability of its three-dimensional configuration. Crystal structures have been solved to date only for the enzyme of the bacterial pathogens *Streptococcus pyogenes* and *Neisseria meningitides* (Nocek et al., 2005) and for the human isozyme 1 (Meng et al., 2006). The plant and the bacterial sequences show similarity over their entire lengths, a fact that is suggestive of a similar tertiary structure. This notwithstanding, an alignment of the deduced amino acid sequences of *S. pyogenes* and *A. thaliana* P5C reductase pointed out a moderate degree of conservation, with 33% identities, 56% conserved residues, and 3% gaps (Forlani et al., 2007). Consistently, the sensitivity of the bacterial enzyme to a group of aminobisphosphonate inhibitors (Forlani et al., 2012) was found to be strikingly higher than that of the plant enzyme (Forlani et al., 2013), showing IC_50_ values 2-3 orders of magnitude lower. Therefore, significant differences may exist with respect to the substrate- and effector-binding protein domains. Moreover, a broad range of oligomeric states of P5C reductase has been reported to date, extending from 125 kDa (suggesting tetramer) to 200–340 kDa (octamer–dodecamer; references in Nocek et al., 2005). For both the *S. pyogenes* (Nocek et al., 2005) and the human (Meng et al., 2006) enzyme a decameric architecture with five homodimer subunits and ten catalytic sites arranged around a peripheral circular groove has been described. Data obtained by gel permeation chromatography for plant P5C reductases were compatible with either a decameric (Murahama et al., 2001) or a dodecameric assembly (Giberti et al., 2014).

In the frame of a research project for integrated genetic and genomic approaches for new Italian rice breeding strategies, we aim at a better understanding of the biochemical mechanisms underlying salt tolerance and proline accumulation in rice. Here we describe the functional characterization of rice P5C reductase. Our results confirmed in a monocotyledonous species the regulatory pattern previously found in *A. thaliana*. Taking one step further, the kinetic mechanisms for product inhibition were elucidated, and the regulatory effects of anions and cations were differentiated. On the whole, these results suggest that under physiological conditions only NADPH would act *in vivo* as the electron donor, and that a stress-induced increase in the cytosolic cation content and/or in the NADPH/NADP^+^ ratio would instantly enhance P5C reductase activity, with no need of transcriptional control. A three-dimensional structure of rice P5C reductase was also obtained, showing a homodecameric configuration.

## Materials and Methods

### Cloning and Heterologous Expression

The coding sequence of *Oryza sativa* P5C reductase was amplified by PCR from cDNA clone J013104L18 (Rice Genome Resource Center, National Institute of Agrobiological Sciences DNA Bank, Japan) with the primers P5CR-fw (caccATGGCGGCGCCGCCTCA) and P5CR-rev (gaggaTTAACTCTGAGAAAG), and inserted into the expression vector pET151 by directional TOPO cloning (Life Technologies, Carlsbad, CA, USA), yielding the vector pET151–*Os*P5CR. For heterologous expression, *E. coli* BL21(DE3) pLysS cells (Invitrogen) were made competent by the calcium chloride method, transformed with the vector and selected on ampicillin-containing LB plates. After inducing the expression of P5C reductase by 1 mM isopropyl-D-thiogalactopyranoside (IPTG) at 24°C, the cells were lysed in a mortar with 2 g g^-1^ alumina and resuspended in 20 mL g^-1^ extraction buffer (50 mM Na phosphate buffer, pH 7.5, containing 200 mM NaCl, 0.5 mM DTT, and 20 mM imidazole). The His-tagged protein was purified from clarified extracts by affinity chromatography with a His-Select^TM^ Nickel Affinity Gel column (1.5 mL bed volume, Sigma H7788). Stepwise elution was achieved by increasing concentrations of imidazole in extraction buffer. For activity assays, the purified enzyme was diluted 1:1000 with water, and a proper aliquot (2–5 μL) was added to the assay mixture. To remove the His-tag, aliquots (100 μg) of the preparation were treated with 1 μg of tobacco etch virus (TEV) protease (Sigma T4455), according to the cleavage protocol provided by the manufacturer.

### Enzyme Assay

The physiological, forward reaction of P5C reductase was measured at 35°C following the P5C-dependent oxidation of NAD(P)H. Unless otherwise specified, the assay mixture contained 20 mM Tris-HCl buffer, pH 7.75, 1 mM NADH or 0.5 mM NADPH, and 1 mM DL-P5C (equivalent to 0.5 mM L-P5C; Williams and Frank, 1975) in a final volume of 0.2 mL. DL-P5C was synthesized by the periodate oxidation of δ-allo-hydroxylysine (Sigma H0377) and purified by cation-exchange chromatography, as described previously (Forlani et al., 1997). A limiting amount of enzyme (from 8 to 16 ng of the purified protein) was added to the pre-warmed mixture, and the decrease in absorbance at 340 nm was recorded at 20-s intervals and up to 5 min through an optical path of 0.5 cm. Activity was calculated from the initial linear rate on the assumption of a molar extinction coefficient for NAD(P)H of 6,220 M^-1^ cm^-1^. Linear regression analysis was computed by using Prism 6 (version 6.03, GraphPad Software, Inc., USA). Protein content was determined by the Coomassie Blue method (Bradford, 1976), using bovine serum albumin (BSA) as the standard. For the purified protein, direct absorbance at 280 nm was used instead, and the concentration was calculated on the basis of a deduced molar extinction coefficient for rice P5C reductase of 14,000 M^-1^ cm^-1^ (http://web.expasy.org/cgi-bin/protparam/protparam).

### Kinetic Analyses

To evaluate substrate affinity, invariable substrates were fixed at the same levels as in the standard assay. The concentration of L-P5C ranged from 150 to 500 μM with NADH as the electron donor, and from 100 to 225 μM with NADPH. The concentration of NADH and NADPH ranged from 50 to 350 μM. To evaluate the mechanism of the inhibition brought about by proline and NADP^+^ on the NADH-dependent activity, NADH concentration ranged from 100 to 800 μM. When evaluating the effect of ions, L-P5C concentration was reduced to 200 μM to minimize the carry-over of chloride anions. All assays were performed in triplicate. *K*_M_ and *V*_max_ values, as well as the concentrations causing 50% inhibition (IC_50_) or 50% stimulation of P5C reductase activity, *K*_I_ values and their confidence intervals were estimated by non-linear regression analysis using Prism 6. Catalytic constants were calculated from *V*_max_ values taking into account a homodecameric composition of the native holoenzyme, having each monomer a molecular mass of 29,670 Da.

### Determination of Isoelectric Point, Native, and Denatured Molecular Mass

Discontinuous SDS–polyacrylamide gel electrophoresis was performed at 20°C by the method of Laemmli with a 4% stacking and a 12% separating gel, using a Minigel system (BioRad). Samples were denatured by boiling 5 min in 62 mM Tris–HCl buffer (pH 6.8), containing 2% (w/v) SDS, 10% (v/v) glycerol, and 5% (v/v) β-mercaptoethanol. Proteins were visualized after staining with 0.1% Coomassie brilliant blue R-250.

Gel permeation chromatography was performed by injecting 100 μL aliquots of the purified protein onto a Superose 12 HR 10/30 (Pharmacia) column that had been equilibrated with 50 mM Tris–HCl buffer, pH 7.75, containing 250 mM NaCl. Elution proceeded at the constant flow of 0.5 mL min^-1^, for the collection of 0.5-mL fractions, while monitoring the eluate at 280 nm (HPLC Detector 432, Kontron). Molecular weight markers for column calibration (Pharmacia) were bovine thyroid thyroglobulin (669 kDa), horse spleen ferritin (440 and 960 kDa), bovine liver catalase (232 kDa), rabbit muscle aldolase (158 kDa), and BSA (67 and 268 kDa). Three runs were carried out for each marker, and six runs for the purified protein.

Isoelectric focusing was performed as described previously (Forlani et al., 1997), with ampholytes within the pH 3.5–10 range (Pharmacia); pI markers (Sigma) were bovine milk β-lactoglobulin A (pI 5.1), bovine erythrocyte carbonic anhydrase II (5.4 and 5.9), and bovine erythrocyte carbonic anhydrase I (6.6). After the run, individual tracks were cut from the gel and either sliced in 5-mm segments for the determination of pH, or stained for protein as above.

### Protein Production for Crystallization

The coding sequence of *O. sativa* P5C reductase was subcloned into vector pMCSG68 according to the standard protocol described previously (Eschenfeldt et al., 2013). *OsP5CR* was overexpressed in BL21 Gold *E. coli* cells (Agilent Technologies). The bacteria were cultured with shaking at 210 rpm in Lysogeny Broth supplemented with 150 μg mL^-1^ ampicillin at 37°C until the OD_600_ reached 1.0. The temperature was lowered to 18°C and IPTG was added to a final concentration of 0.5 mM. The culture was grown for 18 h and the cells were pelleted by centrifugation at 4°C. Bacteria from 1 L culture were resuspended in 35 mL of binding buffer [50 mM Tris-HCl pH 8.0, 500 mM NaCl, 20 mM imidazole, 1 mM Tris(2-carboxyethyl)phosphine (TCEP)] and stored at -80°C. The samples were thawed and the cells were disrupted by sonication using bursts of total duration of 5 min, with appropriate intervals for cooling. Cell debris was pelleted by centrifugation at 18000 *g* for 30 min at 4°C. The supernatant was applied to a column packed with 8 mL of HisTrap HP resin (GE Healthcare) connected to VacMan (Promega), and the chromatographic process was accelerated with a vacuum pump. After binding, the column was washed five times with 40 mL of binding buffer, and the His_6_-tagged protein was eluted with 20 mL of elution buffer (50 mM Tris-HCl pH 8.0, 500 mM NaCl, 300 mM imidazole, 1 mM TCEP). TEV protease (2 mg) was added to cleave the His_6_-tag, and the sample was immediately transferred to a dialysis tube. The dialysis was carried out overnight at 4°C against buffer lacking imidazole. The solution was again mixed with HisTrap HP resin to remove the His_6_-tag and the His_6_-tagged TEV protease. The flow-through, containing rice P5C reductase, was concentrated to 4 mL and applied onto a HiLoad Superdex 200 16/60 column (GE Healthcare) equilibrated with 50 mM Tris-HCl buffer, pH 8.0, containing 200 mM NaCl and 1 mM TCEP. The size exclusion chromatography yielded a homogenous protein fraction.

### Crystallization, Data Collection, and Structure Solution

The sample was concentrated using Amicon concentrators (Millipore) to 14 mg mL^-1^ as determined by measuring the absorbance at 280 nm. Crystallization screening was performed using a Robotic Sitting Drop Vapor Diffusion setup (Mosquito). Manual optimization using hanging drops gave the following final conditions: 100 mM Tris-HCl, pH 8.5, 200 mM MgCl_2_, 18% polyethylene glycol 8000. The crystallization drop was composed of 4 μL of protein and 2 μL of the reservoir solution. The needle-shaped crystals appeared after 3 days at 19°C. The crystals were washed with the reservoir solution supplemented with 20% glycerol as a cryo-protectant and vitrified in liquid nitrogen.

The diffraction data were collected at 22-ID SER-CAT beamline at Advanced Photon Source, Argonne, USA. The diffraction images were processed with XDS (Kabsch, 2010). The structure was solved by molecular replacement in Phaser (McCoy et al., 2007) using a homology-based model of rice P5C reductase prepared using Swiss-Model server (Biasini et al., 2014). The structure of human homolog withdrawn from Protein Data Bank (PDB, access ID: 2izz) served as the template. The protein model was built using Phenix AutoBuild (Terwilliger et al., 2008). Statistics of data collection, processing, and refinement are summarized in **Table 1**.

**Table 1:** Data collection statistics.

Data collection		Refinement	
Wavelength (Å)	0.97625	*R*_free_ reflections	816
Temperature (K)	100	Number of atoms (non-H)	38743
Space group	*P*2_1_	*R*_work_/*R*_free_ (%)	24.6/33.8
Unit cell parameters *a,b,c* (Å) β (°)	84.6, 192.4, 212.1 96.6	RMSD from ideal geometry	
Oscillation range (°)	0.3	Bond lengths (Å)	0.018
Number of images	330	Bond angles (^o^)	2.1
Resolution (Å)	40.0–3.40 (3.60–3.40)	Ramachandran statistics (%)	
Reflections collected/unique	195821/90887	Favored	90.6
Completeness (%)	96.2 (93.7)	Allowed	6.6
Multiplicity	2.1 (2.1)	Outliers	2.8
*R*_meas_^a^ (%)	14.7 (77.0)		
<*I*/σ(*I)*>	9.2 (2.0)		

*Values in parentheses correspond to the highest resolution shell. ^a^*R*_meas_ = redundancy independent R-factor (Diederichs and Karplus, 1997).*

## Results

### Functional Properties of Rice P5C Reductase

The cDNA of the only gene coding for P5C reductase in the *japonica* rice genome was subcloned into the expression vector pET151 and expressed in *E. coli*. The N-terminal addition of a stretch of six His residues (Supplementary Figure S1) and the adoption of a stepwise elution protocol allowed the attainment of homogeneous preparations in a single step (Supplementary Figure S2). The presence of the His_6_-tag did not affect the enzymatic activity, since virtually identical results were obtained in all experiments before and after the cleavage of the purified protein with TEV protease. Enzyme preparations were highly stable; if sterilized by filtration (0.22 μm pore size), no detectable loss of activity was evident after 3-month storage at 4°C.

**FIGURE 1:**
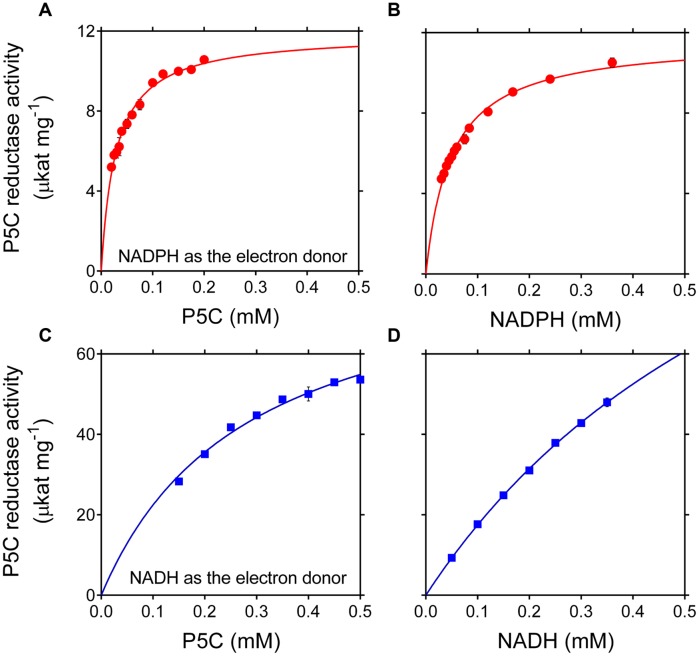
**Effect of substrate concentration on the activity of rice P5C reductase.** The activity of purified recombinant rice enzyme (8 ng) was measured for up to 5 min at 35°C in the presence of increasing concentrations of L-P5C **(A,C)**, NADPH **(B),** or NADH **(D)**. Invariable substrates were fixed at the same levels as in the standard assay. At least three replicates were carried out for each concentration, and mean values ± SE are presented. Plotting of data from Michaelis–Menten graphs into the Lineweaver–Burk double reciprocal plots allowed the calculation of affinity constants and V_max_ values for the NADH- and the NADPH-dependent reaction (**Table 2**).

The maximal specific activity of the recombinant enzyme strongly depended on the electron donor used. With NADPH, a V_max_ of about 12 μmol s^-1^ (mg protein) ^-1^ was found, corresponding to a catalytic constant of 350 s^-1^ per monomer (**Table 2**). With NADH instead of NADPH, a strikingly higher V_max_ value was calculated, one that would result in more than 4,500 catalytic events in 1 s for a single subunit. However, the corresponding affinity was conversely lower (apparent *K*_M_ values of 49 and 806 μM for NADPH or NADH, respectively), and saturating conditions were not obtained even at the highest NADH concentration tested (**Figure 1**). As a consequence, the estimated V_max_ with P5C as the variable substrate was lower than that with NADH (**Table 2**), because the latter was still limiting under standard assay conditions. The use of NADH as the co-substrate also resulted in a 10-fold higher apparent K_M_ value for P5C.

**Table 2:** Properties of rice P5C reductase.

Denatured molecular mass (by SDS-PAGE)	30.1 ± 0.4 kDa
Native molecular mass (by gel permeation)	401 ± 19 kDa
Isoelectric point	6.58 ± 0.04
pH optimum	8.75 ± 0.21
V_Max (NADH)_	158.3 ± 13.2 μkat (mg protein)^-1^
V_Max (P5C, with NADH as the co-substrate)_	85.95 ± 3.53 μkat (mg protein)^-1^
V_Max (NADPH)_	11.68 ± 0.16 μkat (mg protein)^-1^
V_Max (P5C, with NADPH as the co-substrate)_	11.88 ± 0.21 μkat (mg protein)^-1^
K_cat (NADH)_ *per* monomer	4,697 s^-1^
K_cat (NADPH)_ *per* monomer	350 s^-1^
K_M(app)_ for L-P5C _(NADH)_	283 ± 25 μM
K_M(app)_ for L-P5C _(NADPH)_	28.8 ± 1.7 μM
K_M(app)_ for NADH	806 ± 89 μM
K_M(app)_ for NADPH	49.4 ± 1.8 μM

**FIGURE 2:**
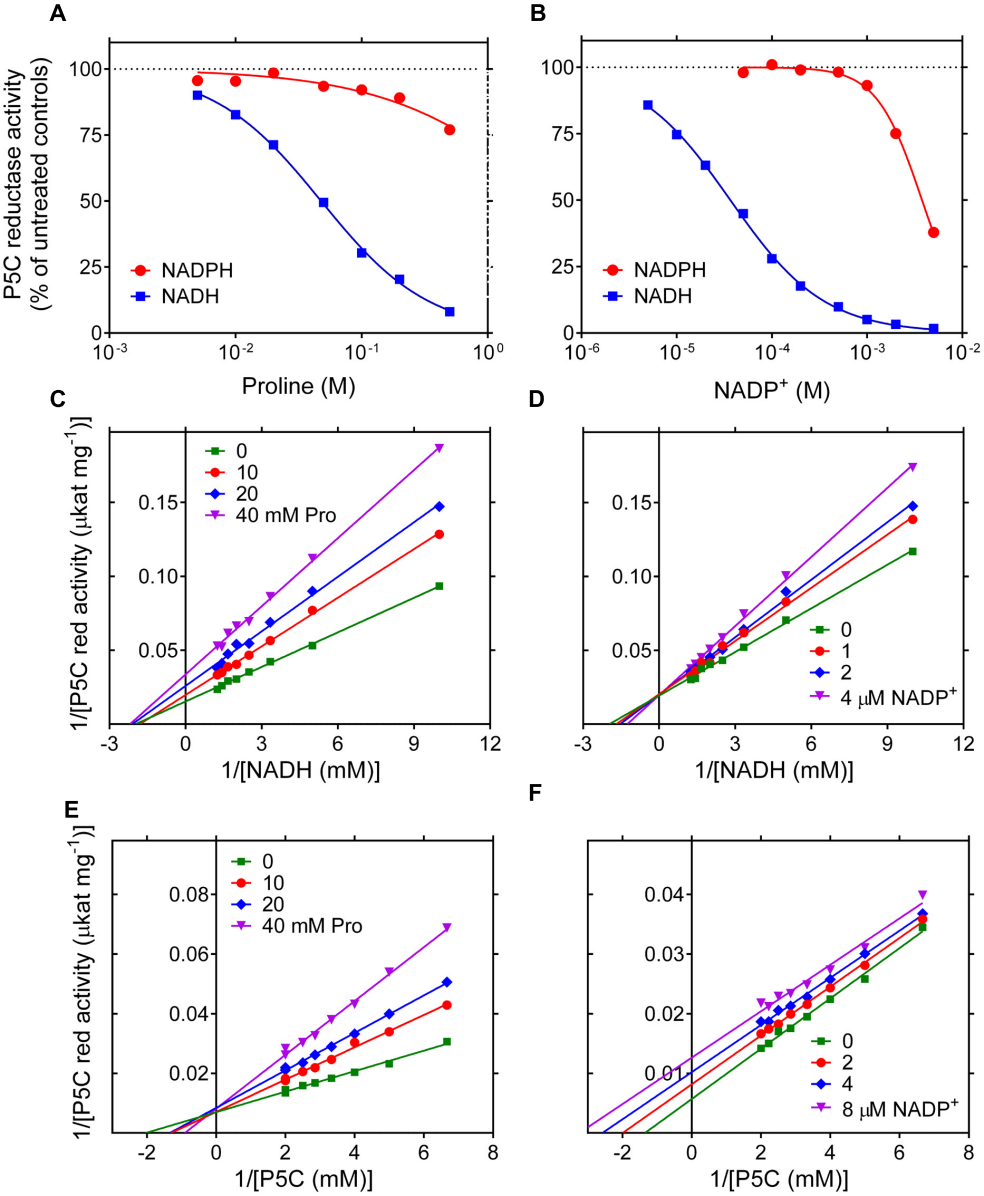
**Product inhibition of rice P5C reductase.** The effect of increasing concentrations of proline **(A)** and NADP^+^ **(B)** on the activity of the enzyme was determined using either NADH or NADPH as the electron donor. Results were expressed as percent of the activity in the absence of supplements, and are mean ± SE over three replicates. Non-linear regression analysis allowed the calculation of IC_50_ values, which in the case of the NADH-dependent reaction were 47.7 ± 1.6 mM and 36.7 ± 1.1 μM for proline and NADP^+^, respectively. The NADPH-dependent activity was much less sensitive to proline and NADP^+^, with estimated IC_50_ values of 3.44 ± 1.51 M and 3.78 ± 0.12 mM, respectively. To investigate the mechanisms of product inhibition, the affinity toward NADH **(C,D)** or L-P5C **(E,F)** was calculated in the presence of increasing concentrations of L-proline **(C,E)** or NADP^+^ **(D,F)**. Lines converging to the y axis account for a mechanism of competitive type with respect to P5C for the inhibition by proline **(E)** and with respect to NADH by NADP^+^ **(D)**, with *K*_I_ values of 20.2 ± 1.0 mM and 7.73 ± 0.69 μM, respectively. Lines converging to the x axis suggest a mechanism of non-competitive type for proline with respect to NADH **(C)**, *K*_I_ being equal to 34.7 ± 1.2 mM. On the contrary, parallel lines for different NADP^+^ concentrations are consistent with an inhibition of uncompetitive type with respect to P5C **(F)**, with a *K*_I_ value of 8.48 ± 0.56 μM.

While the NADPH-dependent activity was linear with time over the entire assay period, kinetics with NADH showed a progressive reduction of the catalytic rate as the reaction took place (data not shown). To verify whether this effect may be due to product inhibition, the impact of increasing levels of proline, NADP^+^ and NAD^+^ on the initial reaction velocity was assessed. In the range of concentrations tested, NAD^+^ was substantially ineffective (results not presented). On the contrary, both proline and NADP^+^ were able to inhibit the activity of P5C reductase (**Figures 2A,B**). However, a remarkably different sensitivity was found depending on the pyridine nucleotide used. With NADPH as the electron donor, proline was inhibitory only at concentrations exceeding 100 mM, and NADP^+^ was effective if added at levels higher than that of NADPH in the assay mixture. On the contrary, the NADH-dependent activity was strongly reduced by the presence of either proline concentrations in the range from 5 to 100 mM, or micromolar levels of NADP^+^, with IC_50_ values of 48 mM and 37 μM, respectively. To obtain further information, a thorough kinetic analysis was performed. When the effects of proline and NADP^+^ were evaluated at varying the concentration of either substrate, Lineweaver-Burk plots were consistent with an inhibition mechanism of competitive type with respect to P5C for proline (**Figure 2E**) and with respect to NADH for NADP^+^ (**Figure 2D**), and of non-competitive type for proline with respect to NADH (**Figure 2C**). Interestingly, for NADP^+^ an inhibition of uncompetitive type with respect to P5C was found (**Figure 2F**), showing that P5C binding is required to allow NADH to enter the active site of the enzyme. *K*_I_ values with respect to NADH were 20 mM and 8 μM for proline and NADP^+^, respectively.

### Effects of Anions and Cations on the Activity of Rice P5C Reductase

**FIGURE 3:**
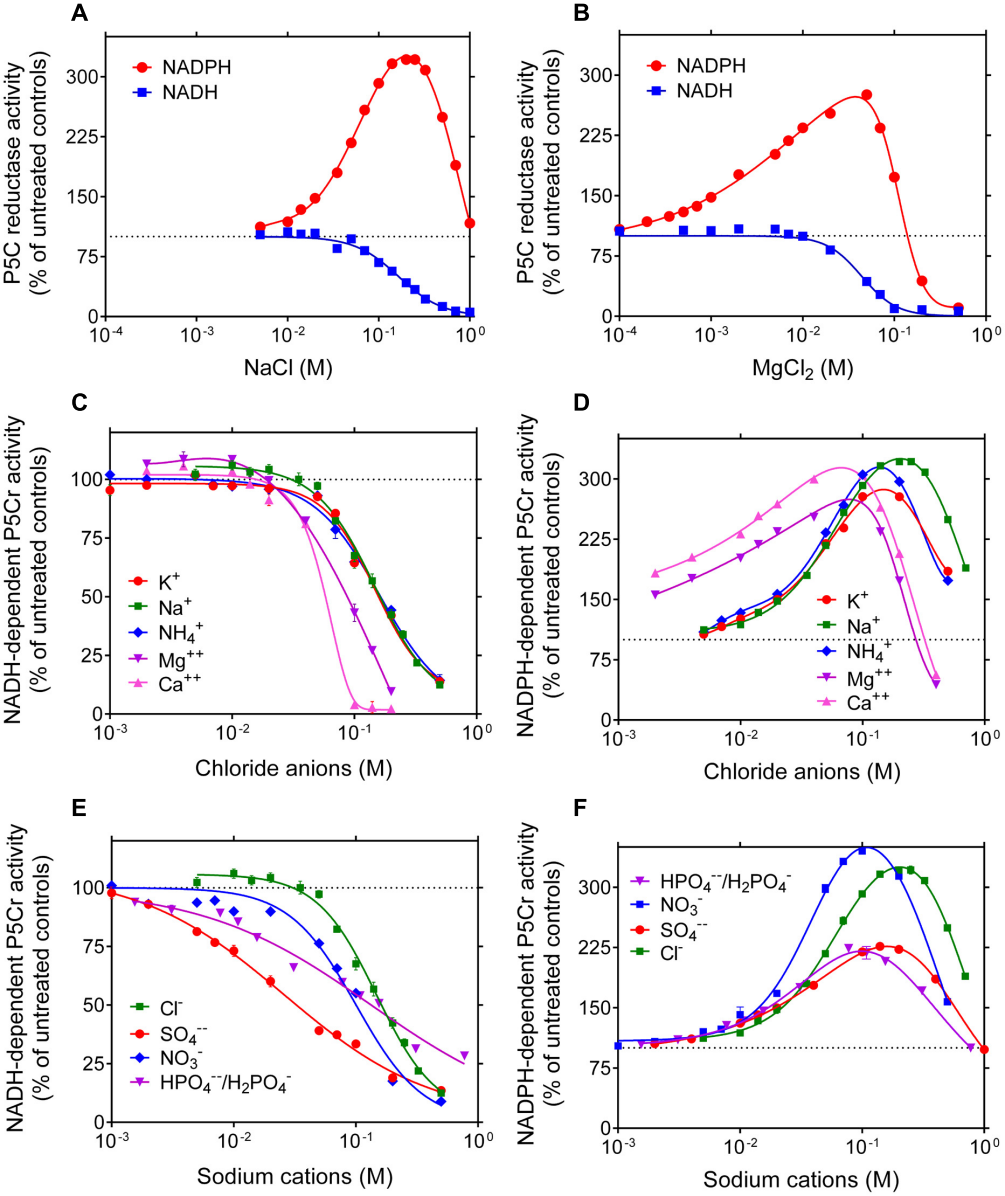
**Effect of anions and cations on the activity of rice P5C reductase.** The effects of the addition of increasing concentrations of NaCl **(A)** or MgCl_2_ **(B)** to the reaction mixture were assessed using either 1 mM NADH or 0.5 mM NADPH as the electron donor. To minimize the carry-over of chloride anions from the purified preparation of the co-substrate, L-P5C levels were fixed at 0.2 mM, resulting in less than 10 mM Cl^-1^ in standard mixture. Results were expressed as percent of controls assayed in the absence of added salts, and are mean ± SE over three replicates. Non-linear regression analysis allowed the calculation of IC_50_ values for the NADH-dependent reaction, which were 165 ± 13 mM and 43.8 ± 3.9 mM for NaCl and MgCl_2_, respectively. To discriminate whether anions or cations were causing the striking stimulation of the NADPH-dependent activity and the inhibition of the NADH-dependent reaction, similar experiments were also performed with increasing concentrations of KCl, NH_4_Cl, and CaCl_2_ **(C,D)**, or with NaNO_3_, Na_2_SO_4_, and NaH_2_PO_4_/Na_2_HPO_4_ (in a molar ratio 0.292: 1, resulting in a pH value of 7.75) **(E,F)**. Results were plotted together as a function of chloride or sodium ion concentration, respectively. Almost overlapping patterns suggest that cations in the range 10^-3^–10^-1^ M stimulate P5C reductase activity when it uses NADPH as the substrate, whereas anions inhibit the reaction if NADH acts as the electron donor.

Because inconsistent results have been described in the literature about the sensitivity of plant P5C reductase to salts, the effect of increasing concentrations of NaCl and MgCl_2_ on the catalytic rate of the rice enzyme was assessed. Once again, strikingly different properties were evident depending on the electron donor (**Figures 3A,B**). With NADH as the co-factor, an inhibition was found with both salts at levels exceeding 20–50 mM. With NADPH, a remarkable stimulation was on the contrary shown at concentrations in the range 5–200 mM NaCl and 0.1–70 mM MgCl_2_. Above these thresholds, the activity came back to control rates and, only in the case of MgCl_2_, was inhibited at levels exceeding 100 mM. To ascertain whether these effects may be ascribed to anions or cations, the effects of other chlorides or other sodium salts were also investigated. Results were plotted as a function of Cl^-^ (**Figures 3C,D**) or Na^+^ (**Figures 3E,F**) concentration when NADH (**Figures 3C,E**) or NADPH (**Figures 3D,F**) was used as the co-factor. Almost overlapping patterns for several chlorides and dissimilar patterns for various sodium salts suggest that anions are the main cause of the inhibition of the NADH-dependent activity. Conversely, different patterns with different chlorides but substantially overlapping patterns with various sodium salts were consistent with a stimulation of the NADPH-dependent activity by cations, whereby divalent cations were remarkably more effective than monovalent cations (**Figure 3D**).

**FIGURE 4:**
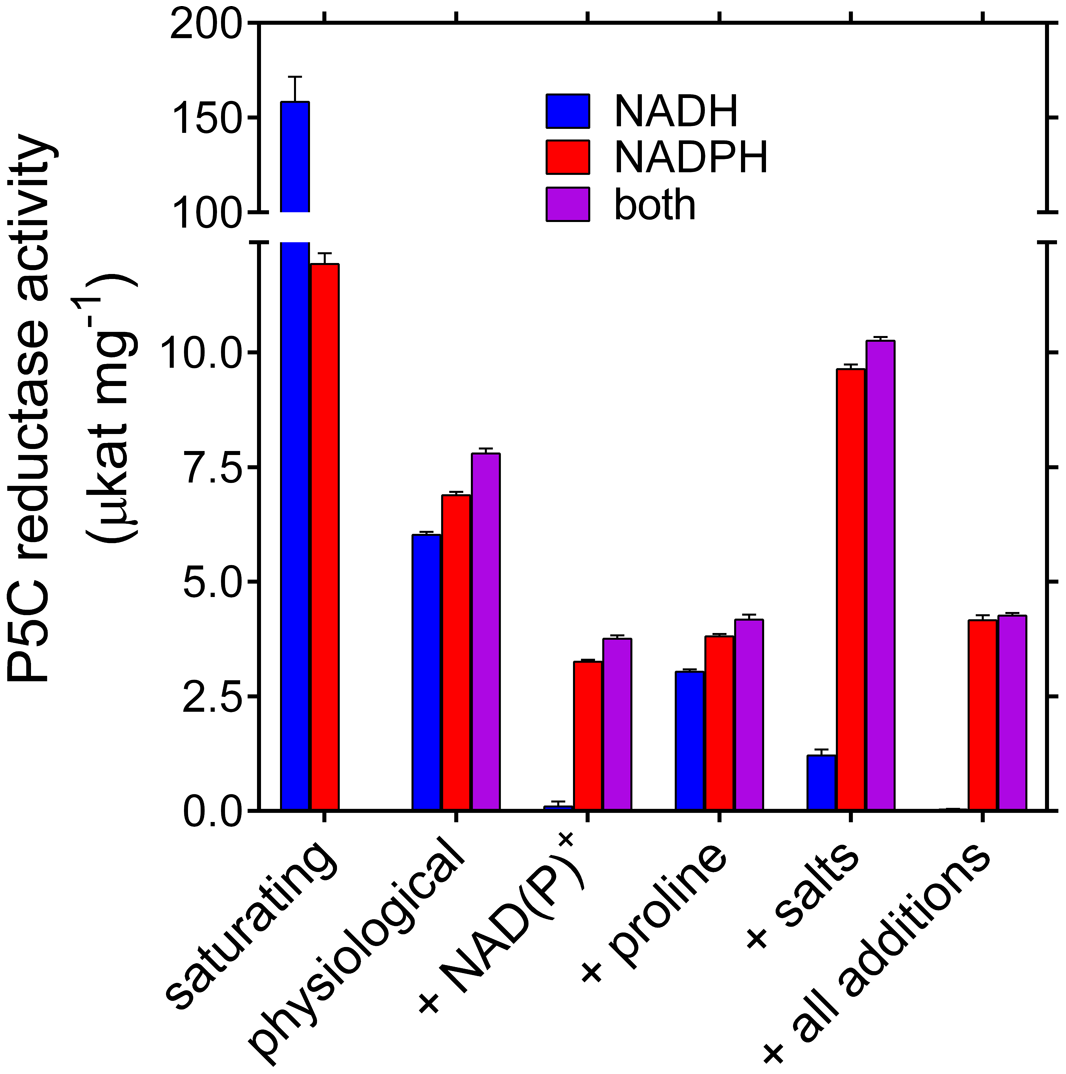
**Activity of rice P5C reductase in the presence of physiological concentrations of substrates, products, and ions.** Specific activity levels of the purified enzyme were measured under conditions simulating substrate and effector levels inside the plant cell. Available literature data in nmol (g fresh weight)^-1^ were converted into molar concentrations by assuming that in cultured plant cells the cytosol may account for about 10% of fresh weight. Activity assays were therefore carried out in the presence of the following concentrations: NADPH 50 μM, NADH 30 μM, NADP^+^ 250 μM, NAD^+^ 160 μM (Hayashi et al., 2005); L-P5C 100 μM (Forlani et al., 2013); Pro 15 mM (Forlani et al., 2015a); 5 mM NaCl, 20 mM KH_2_PO_4_/K_2_HPO_4_, 20 mM K_2_SO_4_, 5 mM NH_4_NO_3_, and 5 mM MgSO_4_, resulting in 76 mM K^+^, 25 mM SO_4_^2-^, 20 mM H_2_PO_4_^-^/HPO_4_^2-^, 5 mM Na^+^, 5 mM Mg^2+^, 5 mM NH_4_^+^, 5 mM NO_3_^-^, and 5 mM Cl^-^ (Lutts et al., 1996; Taiz and Zeiger, 2010). Presented values are means ± SE over eight replicates; activities under saturating substrate conditions are quoted from **Table 2**.

Based on these data, P5C reductase activity seems therefore strongly dependent on both the use of NADH vs. NADPH as the electron donor, and the presence of reaction products and salts. To obtain further information, the activity of the purified enzyme was measured in the presence of substrate and effector concentrations similar to those reported to exist within plant cells. Results are summarized in **Figure 4**. Despite the fact that V_max_ with NADH is more than 10-fold higher than that with NADPH, very similar rates were obtained with either co-factor added to the reaction mixture at physiological levels. The activities were not additive, suggesting a preferential use of NADPH. This notwithstanding, the rate in the presence of both dinucleotides was slightly higher than that with NADPH alone, showing that also NADH may contribute to the overall velocity. However, when realistic levels of NADP^+^ were also present, the NADPH-dependent activity was reduced to 50%, whereas the NADH-dependent reaction was almost abolished. The presence of 15 mM proline was more inhibitory than previously observed under standard assay conditions, most likely because of the lower concentration of P5C. Interestingly, the presence of ion concentrations similar to those reported in the cell under normo-osmotic conditions enhanced the NADPH-dependent activity while inhibiting that with NADH. When all variables were included, the enzymatic activity with NADPH corresponded to about 40% of the maximal rate, whereas that with NADH was completely abolished. Consistently, the activity level with both nucleotides was under these conditions not significantly different from that with NADPH alone.

### Structural Characterization of Rice P5C Reductase

**FIGURE 5:**
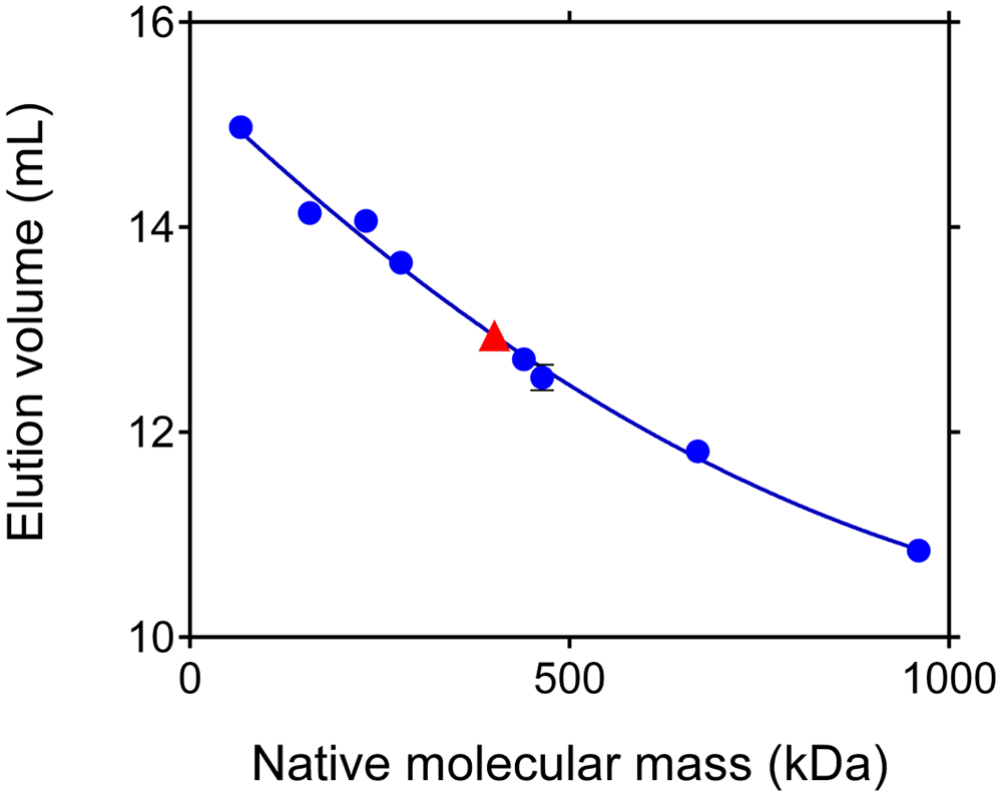
**Apparent molecular mass of rice P5C reductase under native conditions.** Aliquots (100 μL) of the purified protein, adjusted to 10 mg mL^-1^, were subjected to gel permeation chromatography on a Superose 12 HR 10/30 (Pharmacia) column that had been equilibrated with 50 mM Tris–HCl buffer, pH 7.75, containing 250 mM NaCl. A molecular mass of 401 ± 19 kDa was estimated for P5C reductase. Based upon these results, the enzyme could consist of 13–14 subunits. Identical elution patterns were obtained at increasing NaCl concentration up to 1 M (data not shown).

Under denaturing conditions, P5C reductase migrated as a single band (Supplementary Figure S2) to a position corresponding to a molecular mass (30.1 ± 0.4 kDa) that is compatible with the deduced mass from the nucleotide sequence of the gene (28,624 Da; Supplementary Figure S1). To obtain an estimate of its relative mass under non-denaturing conditions, the TEV-cleaved protein was subjected to gel filtration chromatography, and its retention pattern was compared with that of molecular weight markers (**Figure 5**). Results indicated a native molecular mass of 401 ± 19 kDa, which would be consistent with an oligomer composed of 14 identical subunits. However, void volumes within the protein structure as well as any deviation from globular shape strongly affect protein retention, and molecular masses inferred from retention patterns may be subjected to significant errors (Erickson, 2009).

**FIGURE 6:**
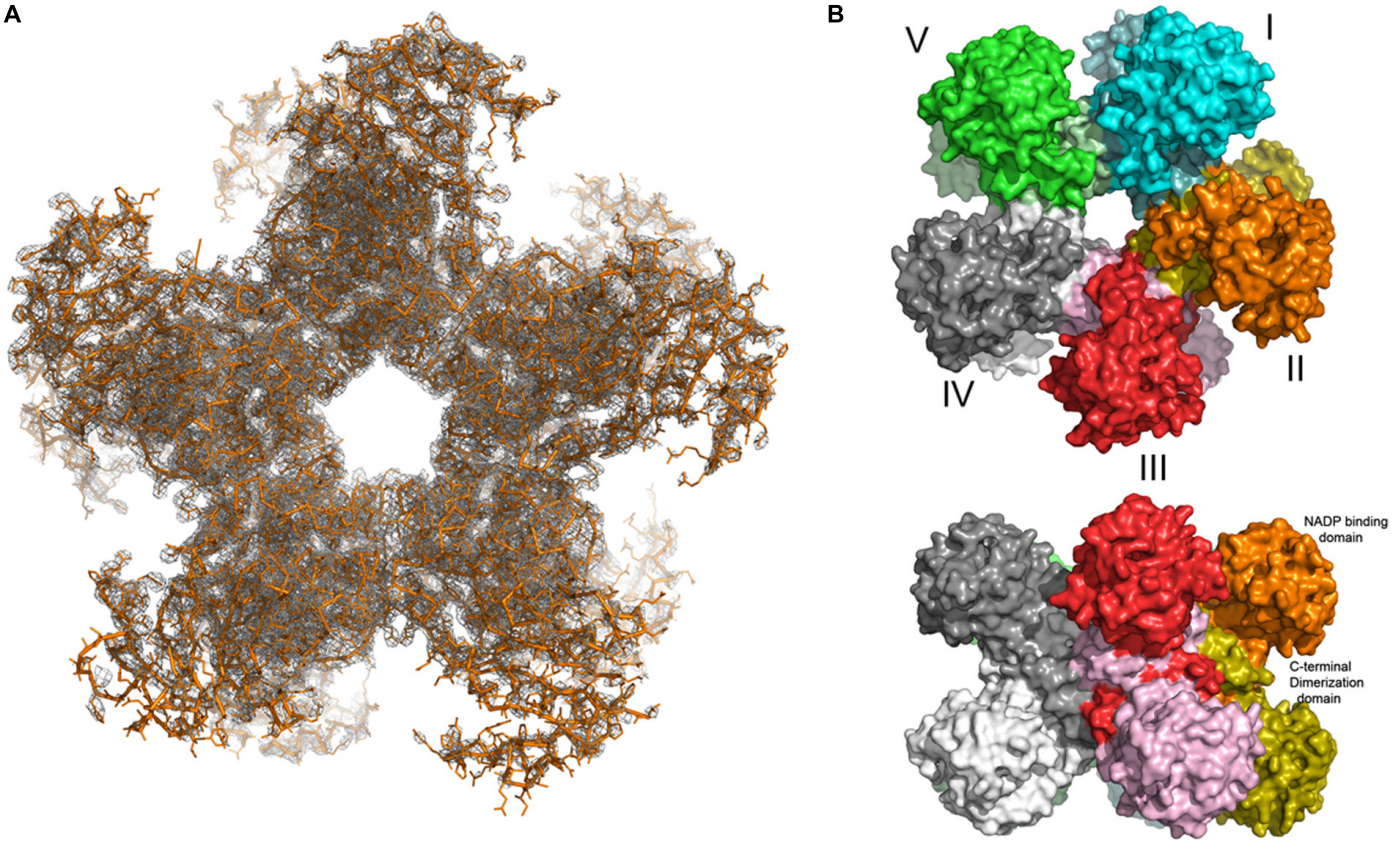
**The decameric structure of of rice P5C reductase.** The top view **(A)** along the fivefold NCS axis reveals a pentamer of dimers, shown in orange. The two *F*_obs_ – *F*_calc_ density maps contour at 1 σ level covering the entire decamer is shown in gray. Top and side views **(B)** of the decamer in surface-representation. The subunits are shown in different colors.

Indeed, a decameric composition was found when the crystal structure of *O. sativa* P5C reductase was determined at a resolution of 3.40 Å. The structure was determined by the molecular replacement method with a search probe created by homology modeling based on the decameric human structure (PDB ID: 2izz; Forlani et al., 2015b). Analysis of the protein crystal solvent content, the so-called Matthew‘s coefficient calculation, indicated that two decameric assemblies are located in the asymmetric unit of the crystal lattice with *P*2_1_ space group symmetry. Rice P5C reductase can therefore be described as a pentamer of dimers, which has a doughnut-like shape with the dimensions of 112 Å × 85 Å (**Figure 6**). This arrangement is formed by a mutual exchange of the C-terminal domains between two neighboring protein subunits, while in contrast the nucleotide binding (N-terminal) domains do not interact with each other and are pointing away from the hollow core. The N-terminal domains are flexible, and only 11 out of 20 (in two decamers) were defined well enough in the electron density maps to be reliably modeled. The hinge that allows for independent movement of the N-terminal domains is predicted to be around the residues 175–180. The protein chain for which the electron density maps were of the best quality was modeled and copied by non-crystallographic symmetry (NCS) operations, and a refinement was performed with secondary structure restraints for all N-terminal domains. This approach allowed us to obtain the model of a full decamer (**Figure 6**). The electron density for nine (out of 20 in two decamers) N-terminal domains was poor, which is evident by the elevated R factors of model R/R_free_ = 25/34%.

## Discussion

### Plant P5C Reductases Share Peculiar Properties that Can be Functional to the Multiple Roles Hypothesized for Proline Metabolism in the Cell

Here we report an exhaustive characterization of rice P5C reductase, affinity-purified after heterologous expression in *E. coli*. Under saturating conditions, the purified enzyme showed a specific activity (about 160 μkat mg^-1^) significantly higher than those of the enzymes isolated from other plant sources [8.5 μkat mg^-1^ for barley (Krueger et al., 1986), 4.4 μkat mg^-1^ for soybean (Chilson et al., 1991) and 5.1–11.3 μkat mg^-1^ for spinach (Murahama et al., 2001)]. However, this exceptionally high turnover rate depended on the use of NADH as the electron donor. With NADPH instead, a V_max_ value of about 12 μkat mg^-1^ was found. In any case, even with NADPH the resulting catalytic efficiency (K_cat_/K_M_ = 7.09 × 10^6^ M^-1^ sec^-1^) is significantly higher than that reported for other enzymes in amino acid metabolism. For instance, a K_cat_/K_M_ = 4.2 × 10^4^ M^-1^ s^-1^ has been found for *E. coli* γ-glutamyl kinase, the enzyme that catalyzes the first step in proline biosynthesis in bacteria (Pérez-Arellano et al., 2006). That of P5C dehydrogenase, the enzyme that oxidizes P5C back to glutamate, ranged from 3.4 × 10^5^ to 4.2 × 10^5^ M^-1^ sec^-1^ in rat (Small and Jones, 1990) and potato (Forlani et al., 1997), respectively. No information is available regarding this parameter for plant P5C reductases other than the similarly high value (26.7 × 10^6^ M^-1^ sec^-1^) found for the enzyme from *A. thaliana* (Giberti et al., 2014). The enzyme from the human pathogen *S. pyogenes* also showed a high K_cat_/K_M_ value (28 × 10^6^ M^-1^ sec^-1^; Petrollino and Forlani, 2012). The extremely high efficiency for the reaction catalyzed by P5C reductase may be a consequence of the need to rapidly convert even small amounts of P5C into proline, in order to avoid possible cytotoxic effects caused by P5C accumulation (Deuschle et al., 2004; Miller et al., 2009; Senthil-Kumar and Mysore, 2012). This seems consistent with the relatively high *K*_M(app)_ values found for L-P5C (**Table 2**), suggesting that the activity of the enzyme is far from being saturated at physiological concentrations of the substrate: any increase in P5C concentration would therefore result in an equivalent increase of the rate of its utilization by P5C reductase.

The significantly higher maximal reaction rate observed with NADH most likely depends on a faster NAD^+^ release from the active site. Consistently, the NADH-dependent activity was strikingly inhibited by micromolar concentrations of NADP^+^. If both pyridine nucleotides were made available, the reaction proceeded at rates only slightly higher than those in the presence of NADPH alone, confirming a preferential use of NADPH but also suggesting that both co-factors could be used alternately. Such a substrate ambiguity, together with the inhibition by oxidized pyridine dinucleotides, has been interpreted as functional to the proposed role of proline synthesis in maintaining a favorable NADP^+^/NADPH ratio under stress conditions, as well as in regenerating NADP^+^ in photosynthetic tissues (Sharma et al., 2011). From this perspective, in humans the non-allosterically regulated isozyme specifically expressed in erythrocytes, PYCR1, would serve primarily for NADP^+^ generation, whereas the other, proline-sensitive isozyme ubiquitously expressed in the other tissues would be devoted to proline synthesis (Merrill et al., 1989). Since only a single enzyme form is present in most plants, both functions might be ensured through the emerging complex pattern of substrate preference and product inhibition, where proline and NADP^+^ inhibit only the NADH-dependent reaction.

Patterns in Lineweaver–Burk plots (**Figure 2F**) accounted for an inhibition by NADP^+^ of uncompetitive type with respect to P5C. This strengthens earlier results obtained with phosphonate inhibitors of plant P5C reductase (Forlani et al., 2007, 2008) and provides experimental evidence supporting an ordered substrate binding, previously hypothesized only on the basis of the crystal structure of the bacterial enzyme (Nocek et al., 2005). The present data therefore suggest that P5C binds before NADPH. Since in the case of most NAD(P)H-dependent reductases the coenzyme binds before the substrate (Sanli et al., 2003), this is an unusual feature that might depend on the cyclic structure of both substrate and product.

Most interestingly, the use of either co-factor had drastic effects on the susceptibility of P5C reductase to the presence of salts. A twofold stimulation by 100 mM KCl or 10 mM MgCl_2_ had been reported for the enzyme partially purified from pea (Rayapati et al., 1989), but in that case activity had been evaluated with NADH as the electron donor. Conversely, the two isozymes purified from spinach were inhibited by NaCl in the 100–500 mM range, and by MgCl_2_ at lower concentrations, when assayed using NADPH (Murahama et al., 2001). These contrasting results could imply a functional diversity among plant P5C reductases. However, the experiments performed in this study on the enzyme from rice, a monocot, yielded patterns overall similar to those previously obtained with the enzyme from the dicot *A. thaliana* (Giberti et al., 2014). Therefore, it appears more likely that such differences may depend on non-uniform experimental conditions, e.g., the inclusion of different MgCl_2_ levels in the standard assay mixture. Most importantly, in previous studies the possibility that significant levels of chloride and Na^+^ or K^+^ ions may be present in the standard reaction mixture as a consequence of P5C buffering has been largely underestimated. P5C is a labile compound; when synthesized by the periodate oxidation of hydroxylysine, it is purified by cation-exchange chromatography in 1 M HCl (Williams and Frank, 1975). The resulting low pH values stabilize the compound that is routinely stored under these conditions and neutralized just before the enzymatic assay. If neutralization were achieved with sodium or potassium hydroxide, concentrations as high as 100 mM NaCl or KCl would be present in “untreated controls.” In the present study, the adoption of strictly controlled assay conditions ensured that chloride ion concentration in controls was never higher than 15 mM, and no inorganic cations were present.

Making one step forward, the effect of various cations and anions was investigated. Our results clearly show that anions are the cause of the inhibition of the NADH-dependent reaction of P5C reductase, with sulfates being inhibitory at concentrations as low as 1 mM. On the contrary, the stimulation of the NADPH-dependent activity seems to depend on the presence of cations, and divalent cations were effective at lower doses than monovalent ions (**Figure 3D**). Divalent anions at high concentration had detrimental effects, since the Na^+^-dependent stimulation of enzyme activity was lower with sodium salts of divalent anions than that obtained with sodium salts of monovalent anions (**Figure 3F**). If the concentrations at which these effects were evident *in vitro* are considered, in several cases it seems likely that they can occur *in vivo* and influence the resulting rate of proline synthesis.

### Substrate Affinity, Product Inhibition, and Ion Effects May Unravel Substrate Ambiguity, and Represent a Likely Mechanism for *in vivo* Modulation of P5C Reductase Activity

Taking into account the main, if not exclusive, cytosolic localization of P5C reductase (Funck et al., 2012), the question remains as to which may be the physiological electron donor. The mechanisms for substrate inhibition that have been shown in the present study for the NADH-dependent activity seem to substantially prevent the use of the non-phosphorylated co-factor. Indeed, in the presence of NAD(P)(H) and proline concentrations similar to those reported for rice cells, the activity with both electron donors did not significantly differ from that with NADPH alone, probably because the inhibitory effect of proline was amplified by the low physiological concentration of P5C (Forlani et al., 2013), and that of NADP^+^ by the high NADP^+^/NADH ratio (Hayashi et al., 2005; **Figure 4**). Notwithstanding this, the ability of NADH utilization could be useful in special circumstances.

The activation of proline synthesis may maintain a favorable redox balance inside the cell. On the other side, the complex pattern of co-factor preference, substrate inhibition, and salt effects may contribute to a fast activation of proline synthesis under salt stress conditions. Following the exposure to hyperosmotic stress, the oxidative pentose phosphate pathway (OPPP) is rapidly induced, leading to cytosolic NADPH production (Baxter et al., 2007). Moreover, stress-induced inward Ca^2+^-fluxes are able to activate calmodulin-modulated NAD kinase isozymes (Hashida et al., 2009) that in turn increase the NADP(H)/NAD(H) ratio. A higher NADPH availability would enhance the activity of P5C reductase, which shows an K_M(app)_ for NADPH very close to the intracellular concentration of the dinucleotide, and raise the carbon flux within the proline biosynthetic route. Consistently, *A. thaliana* plants overexpressing NAD kinase 2 showed increased levels of free proline (Takahashi et al., 2009). In the meantime, the activity of the OPPP would lower the NADP^+^/NADH ratio, relieving in part the inhibition of the NADH-dependent activity. Over a longer period, an increase of the cytosolic Na^+^ concentration, which can reach 60 mM (Anil et al., 2007), would enhance further the NADPH-fueled reaction. In this way, P5C reductase would be able to respond to wide fluctuations of P5C synthesis by P5C synthetase isozymes without the need of a transcriptional control.

Also changes in the levels of other ions could modulate P5C reductase activity. For instance, magnesium ion concentration in rice cells is estimated to range between 1 and 10 mM (Hayatsu et al., 2014), and it was found to increase up to three-fold in leaves of salt-stressed rice plants (Bertazzini et al., 2012). Such fluctuations would positively affect the catalytic rate of rice P5C reductase using NADPH as the electron donor. In any case, the overall picture supporting the occurrence of differential effects of salts on the activity of P5C reductase depending on the electron donor used (**Table 3**) allows to explain previous contradictory findings. For instance, the translation inhibition of *AtP5CR* under stress conditions (Hua et al., 2001), and the inhibition of spinach P5C reductase by salts in the 10^-2^–10^-1^ M range (Murahama et al., 2001), results that in the absence of post-translational mechanisms modulating enzyme activity and a strong preference *in vivo* for NADPH, respectively, would be inconsistent with stress-induced proline accumulation.

**Table 3:** Effect of selected factors on the activity of rice P5C reductase depending on whether NADPH or NADH acts as co-substrate.

	With NADH	with NADPH
NAD^+^	⇔	⇔
NADP^+^ (10^-5^–10^-3^ M)	⇩⇩	⇔
Proline (10^-2^–10^-1^ M)	⇩	⇔
Monovalent anions (10^-2^–10^-1^ M)	⇩	⇔
Divalent anions (10^-3^–10^-1^ M)	⇩⇩	⇩
Monovalent cations (10^-2^–10^-1^ M)	⇔	⇧⇧
Divalent cations (10^-3^–10^-1^ M)	⇩	⇧⇧⇧

### The Three-Dimensional Structure of P5C Reductase is Conserved Across all Kingdoms of Life, but the Rice Enzyme Reveals Dynamic Movements

Here, we also report the first crystal structure of a plant P5C reductase. Our analysis was limited to basic structural studies due to the fact that only low-resolution (3.40 Å) data were obtained, and some problems hindered the refinement of the structure influencing the quality of the final model. Due to high R-factors and lack of the electron density for a substantial part of the protein, we decided not to deposit the structure in the PDB. Nevertheless, the structural results are solid enough to solve the uncertainties about the oligomeric state of plant P5C reductase. In previous studies the migration of the native protein during gel permeation chromatography led to a rough estimate of 10–12 monomers in spinach (Murahama et al., 2001), and of 12–14 monomers in *A. thaliana* (Giberti et al., 2014). Also the rice enzyme showed an elution profile that is consistent with a 14-mer (**Figure 5**). Based on the structural information obtained, a decameric arrangement might therefore be assumed for all three plant proteins.

However, even though the bacterial, the human, and the plant enzymes form very similar decameric arrangements, the structure of rice P5C reductase reveals dynamic movements of the domains. During model building and refinement, several of the dinucleotide binding domains had unclear or missing density and could not be reliably modeled, which suggests the presence of mobile elements in the crystal (**Figure 6**). This is contrasting with previous studies on bacterial (*S. pyogenes* and *N. meningitides*) representatives that have shown almost identical conformation of the subunits in their substrate-free and substrate-bound structures. Because no conformational changes were observed in these proteins, it was hypothesized that they operate by the lock and key mechanism (Nocek et al., 2005). Similarly, no significant conformational changes were reported for the extensively studied human enzyme (Meng et al., 2006). One possible explanation of lack of movement could be the formation of crystal lattice contacts between domains and their stabilization. In fact, an inspection of crystal contacts in the case of the human P5C reductase confirmed the presence of interactions between molecules that lock the previously mentioned hinge between the two domains of monomers (Salemme et al., 1988). The presence of a conformational plasticity implies the existence of regulation mechanisms. Many allosteric systems contain semi-rigid domains or subunits interacting via flexible regions. This design allows for the propagation of local events over a long distance to affect activities elsewhere (Cui and Karplus, 2008). Further biochemical and biophysical studies are required to investigate the dynamic nature of P5C reductases. However, it appears that these enzymes might be more dynamic than it has been previously thought.

## Conflict of Interest Statement

The authors declare that the research was conducted in the absence of any commercial or financial relationships that could be construed as a potential conflict of interest.
