# Supplementary material for: Functional properties and structural characterization of rice δ1-pyrroline-5-carboxylate reductase
Source: Front Plant Sci. 2015 Jul 28;6:565. doi: 10.3389/fpls.2015.00565 (PMC4517315; doi:10.3389/fpls.2015.00565)
Supplement: Supplementary file 1 [file Figure_S1.PDF]

```
pET151-OsP5CR      TTTTACTTTAAGAAGGAGATATACATATGCATCATCACCATCACCATGGTAAGCCTATCC

pET151-OsP5CR      CTAACCCTCTCCTCGGTCTCGATTCTACGGAAAACCTGTATTTTCAGGGAATTGATCCCT

pET151-OsP5CR      TCACCATGGCGGCGCCGCCTCAGCCCGTGCCGGCGCCCGCGGCCGCGTCGCCGGAGGTGT
clone J013104L18         ATGGCGGCGCCGCCTCAGCCCGTGCCGGCGCCCGCGGCCGCGTCGCCGGAGGTGT

pET151-OsP5CR      TCCGGCTCGGGTTCATCGGCCCGGGTAACCTCGCGGAGAGCATCGCTCGCGGCGTGGCGG
clone J013104L18   TCCGGCTCGGGTTCATCGGCCCGGGTAACCTCGCGGAGAGCATCGCTCGCGGCGTGGCGG

pET151-OsP5CR      CGTCGGGCGTCCTCCCGGCCACCGCGATCCGCACCGCGCCACACCGCCGCCCCGAGCGCG
clone J013104L18   CGTCGGGCGTCCTCCCGGCCACCGCGATCCGCACCGCGCCACACCGCCGCCCCGAGCGCG

pET151-OsP5CR      CCGAGGCCTTCTCATCCATCGGAGCTCACATCTTGGAGACCAACGCGCAGGTAGTTGATG
clone J013104L18   CCGAGGCCTTCTCATCCATCGGAGCTCACATCTTGGAGACCAACGCGCAGGTAGTTGATG

pET151-OsP5CR      ACAGTGACGTGATCGTCATCTCTGTGAAGCCGCAGATCGTAAGGCAGGTACTGGTTGAGC
clone J013104L18   ACAGTGACGTGATCGTCATCTCTGTGAAGCCGCAGATCGTAAGGCAGGTACTGGTTGAGC

pET151-OsP5CR      TAAAACCCTTGCTGTCAGAAGAAAAGCTTCTGGTGTCCATTGCTGCTGGCATCAAGATGG
clone J013104L18   TAAAACCCTTGCTGTCAGAAGAAAAGCTTCTGGTGTCCATTGCTGCTGGCATCAAGATGG

pET151-OsP5CR      AAGATCTGCAGGGTTGGTCTGGTCATCGAAGATTTATTAGAGTAATGCCAAACACCCCTT
clone J013104L18   AAGATCTGCAGGGTTGGTCTGGTCATCGAAGATTTATTAGAGTAATGCCAAACACCCCTT

pET151-OsP5CR      CAGCTGTTGGACAAGCAGCATCAGTGATGTGTTTGGGGGAGATGGCTACTGAGAATGATG
clone J013104L18   CAGCTGTTGGACAAGCAGCATCAGTGATGTGTTTGGGGGAGATGGCTACTGAGAATGATG

pET151-OsP5CR      AAAACCGTGTAAGAAGTTTATTCAGTGCCATTGGAAAAGTTTGGACAGCTGAAGAAAAAT
clone J013104L18   AAAACCGTGTAAGAAGTTTATTCAGTGCCATTGGAAAAGTTTGGACAGCTGAAGAAAAAT

pET151-OsP5CR      ATTTTGATGCTGTAACTGGGCTAAGTGGTAGTGGCCCGGCTTATATTTTCTTAGCAATAG
clone J013104L18   ATTTTGATGCTGTAACTGGGCTAAGTGGTAGTGGCCCGGCTTATATTTTCTTAGCAATAG

pET151-OsP5CR      AGGCCATGGCTGATGGTGGAGTTGCTGCTGGTCTTCCTCGGGATCTTGCACTTGGTCTTG
clone J013104L18   AGGCCATGGCTGATGGTGGAGTTGCTGCTGGTCTTCCTCGGGATCTTGCACTTGGTCTTG

pET151-OsP5CR      CATCTCAGACAGTTCTTGGTGCTGCAACCATGGTAAACAAGACTGGAAAACATCCGGGTC
clone J013104L18   CATCTCAGACAGTTCTTGGTGCTGCAACCATGGTAAACAAGACTGGAAAACATCCGGGTC

pET151-OsP5CR      AGCTGAAGGATATGGTCACTTCCCCGGCAGGAACCACCATAACTGGGATACAAGAGCTTG
clone J013104L18   AGCTGAAGGATATGGTCACTTCCCCGGCAGGAACCACCATAACTGGGATACAAGAGCTTG

pET151-OsP5CR      AGAAGGGTGCATTCCGTGGGACGCTGATAAATGCCGTTGTTGCTGCTACAAAGCGTTGCC
clone J013104L18   AGAAGGGTGCATTCCGTGGGACGCTGATAAATGCCGTTGTTGCTGCTACAAAGCGTTGCC

pET151-OsP5CR      GTGAACTTTCTCAGAGTTAATCCTCTTGTTANNTGTTAGTCCATTNNATGTGCNACTCTG
clone J013104L18   GTGAACTTTCTCAGAGTTAATCCTCTTGTTAGTTGTTAGTCCATTGGATGTGCGACTCTG
```

ATG start codon   stop codon   Primers (Fwd-Rev)

Translation

```
MHHHHHHGKPIPNPLLGLDSTENLYFQGIDPFTMAAPPQPVPAPAAASPEVFRLGFIGPGNLAESI
ARGVAASGVLPATAIRTAPHRRPERAEAFSSIGAHILETNAQVVDDSDVIVISVKPQIVRQVLVEL
KPLLSEEKLLVSIAAGIKMEDLQGWSGHRRFIRVMPNTPSAVGQAASVMCLGEMATENDENRVRSL
FSAIGKVWTAEEKYFDAVTGLSGSGPAYIFLAIEAMADGGVAAGLPRDLALGLASQTVLGAATMVN
KTGKHPGQLKDMVTSPAGTTITGIQELEKGAFRGTLINAVVAATKRCRELSQS
```

polyhistidine tag   V5 epitope   TEV cleavage site   stabilizer

Supplementary Figure 1. Nucleotide and deduced amino acid sequence of *Oryza sativa* P5C reductase. Upper part: Alignment of the construct for expression of rice P5C reductase in *E. coli* with the native cDNA. Primer sequences are underlined. Lower part: The recombinant protein has 33 extra amino acids, but 27 of them are removed by TEV protease; the remaining residues upstream of start of the native protein are double underlined.
