# Supplementary figures and images for: Functional properties and structural characterization of rice δ1-pyrroline-5-carboxylate reductase
Source: Front Plant Sci. 2015 Jul 28;6:565. doi: 10.3389/fpls.2015.00565 (PMC4517315; doi:10.3389/fpls.2015.00565)

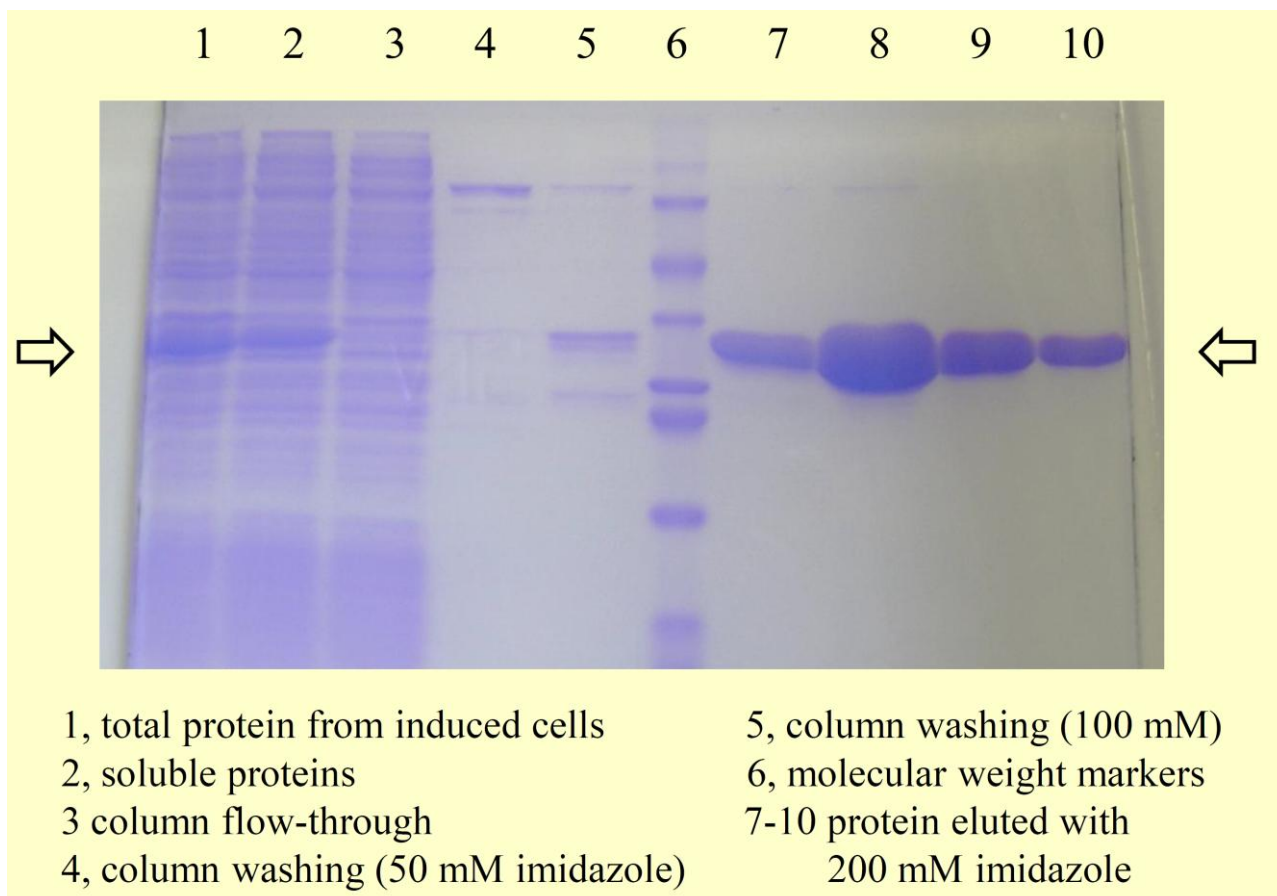

**Supplementary Figure 2.** Affinity purification of *Oryza sativa* P5C reductase expressed in *E. coli*.

Supplement: Supplementary file 2 [file Figure_S2.PDF]
